# How can I explore high-dimensional data?

**DOI:** 10.1590/2176-9451.19.6.014-015.ebo

**Published:** 2014

**Authors:** Glaucia Cristina Rodrigues Nascimento, Marcela Baraúna Magno, Giseon Heo, David Normando

**Affiliations:** 1 Federal University of Pará, Masters student in Dentistry, Federal University of Pará (UFPA); 2 University of Alberta, Canada, Associate professor, University of Alberta, Canada; 3 UFPA, Adjunct professor, UFPA

High-dimensional data hinder sample visualization and limit exploration of data^1^. In these cases, we can make use of multivariate analysis techniques, such as Factor Analysis (FA) and/or Principal Component Analysis (PCA), to reduce a complex data set to one of lower dimensions so as to reveal any hidden features and simplify understanding.

In order to render interpretation of FA and PCA easier, an example of the practical applications of these techniques is described herein.

An orthodontist wants to make a few changes in his clinic in order to ensure higher-quality treatment. However, such changes have to be tailored according to the needs and desires of his target customers. Thus, he decided to implement a questionnaire at the end of orthodontic treatment. Patients had to respond to several questions which were grouped into the following items: 1 - staff helpfulness; 2 - staff professionalism; 3 - staff manners; 4 - attention given by the dentist; 5 - dentist's technical quality; 6 - waiting time; 7 - explanation about treatment; 8 - comfortable facilities; 9 - waiting time to schedule an appointment; 10 - convenience of treatment schedule; 11 - parking facilities; 12 - telephone service; 13 - cleanliness. When rating, patients used a score that ranged between 1 and 7, with 1 meaning weak and 7 excellent. A total of 50 patients responded to the questionnaire. Afterwards, the dentist found it difficult to establish a change plan due to the large number of items analyzed.

In an attempt to reduce the amount of data and facilitate interpretation, the dentist used Factor Analysis (FA), a type of multivariate analysis.* Factor Analysis is useful when there is a large number of variables that may provide redundant or duplicated information. In this case, redundancy means that some variables are correlated to each other - possibly because they are measuring the same "thing". Thus, it is possible to reduce the observed variables to a smaller number also known as factors - groups of correlated variables that present some information in common.*

The orthodontist found out that the 13 items in the questionnaire were not in fact measuring 13 different facts due to the apparent redundancy shared by some variables. Thus, four new variables or factors, which were implicit in the correlations, were established: 1 - efficiency; 2 - comfort; 3 - staff-patient relationship; 4 - dentist-patient relationship. Factor analysis for the variables used by the orthodontist are observed in Figure 1.

**Figure 1.**
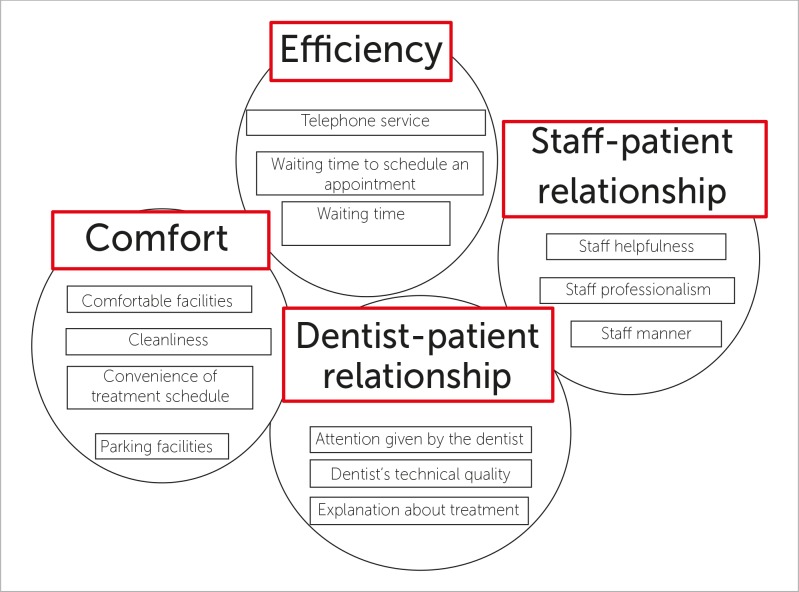
The four factors generated by Factor Analysis.

Nevertheless, to establish an improvement plan, the orthodontist needed to know the item which most influences patients' satisfaction. To this end, he used Principal Component Analysis (PCA), another multivariate analysis employed to explore data.

*PCA is a mathematical tool widely used for high dimensional data analysis.* 2 *It aims at finding the principal components *(PCs). *But what does this mean? Although a large number of variables is necessary to reproduce the overall variability of a particular phenomenon (patients satisfaction, for example), several variables will generally have greater importance than others. PCA identifies these important variables and shows them in decreasing order from the most to the least important. In other words, each new component accounts for progressively smaller amounts of total variance. Importantly, the number of components extracted in PCA is equal to the number of variables being analyzed. In other words, an analysis of the 13-item questionnaire resulted in 13 components. Nevertheless, should more than 80% of variability be explained by a few PCs, we can use them in place of the original variables without losing too much information (this is why only the first components were used for data interpretation).* ^3^

The orthodontist applied PCA over FA for the components previously grouped. Results revealed that dentist-patient relationship and comfort account for 85% of total variance in patient's satisfaction. With these data in hand, the dentist will be able to perform changes in his office and ensure improvements in the items which most influence his target customers.

## PRINCIPAL COMPONENT ANALYSIS IS NOT FACTOR ANALYSIS

Similarly to Factor Analysis (FA), PCA aims to reduce data dimensions to the smallest number of variables possible with minimal loss of information. Nonetheless, there are significant conceptual differences between the two techniques. PCA focuses on summarizing data in order of importance,^3^ whereas FA explains the common variations among the original variables.

Importantly, one should note whether the variables present a connecting structure between them, since both PCA and FA are sensitive to poor correlations established among different variables. This may affect analysis, thereby invalidating the use of these techniques. 

Multivariate analysis has several techniques of different applicability. PCA and FA are two of them, and the choice of which one should be used depends on the hypothesis one intends to generate about the data. Thus, when these multivariate analysis techniques are used properly, they allow us to make inferences beyond the "statistically significant", thereby adding much value to our research. However, it is very important to remember that, in many cases, it may not be easy to interpret principal components and factors, as it depends on the researcher to interpret the new set of variables and be able to translate the information it contains.
